# Association between dietary vitamin B2 intake and constipation: a nationwide cross-sectional study

**DOI:** 10.3389/fmed.2025.1598415

**Published:** 2025-06-23

**Authors:** Chuanyu Ma, Ning Ma, Hongyang Lu, Tianye Huang, Jingtao Zhang, Minglei Luo, Kening Zhang

**Affiliations:** ^1^Department of Proctology, Linyi Central Hospital, Linyi, Shandong, China; ^2^Department of Nutrition and Food Hygiene, School of Public Health, Qingdao University, Qingdao, China

**Keywords:** vitamin B2, riboflavin, constipation, dose-response, NHANES (National Health and Nutrition Examination Survey)

## Abstract

**Background:**

A multitude of studies has suggested a possible link between the intake of dietary micronutrients and the incidence of constipation. Nevertheless, there exists a significant gap in research that specifically addresses the relationship between vitamin B2 and constipation. The present investigation seeks to explore the possible correlation between dietary intake of vitamin B2 and chronic constipation in adult participants surveyed in the National Health and Nutrition Examination Survey (NHANES).

**Methods:**

This research leveraged data from the NHANES conducted between 2005 and 2010. Dietary intake information was obtained from participants through 24 h dietary recall interviews. Various statistical approaches, including weighted logistic regression, interaction tests, subgroup analyses, and restricted cubic spline, were utilized to examine the relationship between dietary vitamin B2 consumption and the occurrence of constipation.

**Results:**

A comprehensive multiple logistic regression analysis that accounted for various confounding factors indicated that individuals with the highest quartile of vitamin B2 intake exhibited a significantly reduced risk of experiencing constipation (OR = 0.63; 95% CI, 0.47–0.84) in comparison to those situated in the lowest quartile. Furthermore, a continuous assessment of vitamin B2 intake demonstrated an inverse relationship with constipation (OR = 0.89, 95% CI, 0.81–0.98). The implementation of restricted cubic splines suggested a linear association between vitamin B2 consumption and constipation (*P*-non-linear = 0.7297, *P*-overall = 0.0224). Notably, subgroup analyses uncovered a significant interaction effect between vitamin B2 intake and alcohol consumption regarding constipation (*P*_*for interaction*_ = 0.012).

**Conclusion:**

This investigation highlights an inverse relationship between the dietary intake of vitamin B2 and constipation within the adult population of the United States.

## 1 Introduction

Constipation is a commonly encountered symptom linked to various chronic diseases, negatively impacting the physiological functions within the human body. The persistence of chronic constipation not only intensifies the burden on healthcare systems but also increases the associated costs of treatment (1, 2). In recent years, shifts in lifestyle and dietary habits have contributed to a notable rise in the prevalence of functional constipation, thereby posing a considerable threat to the health and wellness of those affected. An emerging synthesis of systematic evidence and meta-regression revealed that the worldwide prevalence of constipation is roughly 14% (3). Moreover, chronic constipation can result in several complications, including hemorrhoids, intestinal obstruction, and anal fissures, which place significant demands on healthcare resources and public health systems (4, 5).

While constipation pathogenesis involves multiple determinants, nutritional components remain a well-characterized modulator in its development. Studies indicate that specific dietary components, including soluble fiber and essential trace elements like vitamin B1, vitamin E, magnesium, selenium, and phosphorus (6–10), may contribute to mitigating the risk of chronic constipation. B vitamins, as water-soluble micronutrients, serve as enzymatic cofactors mediating fundamental biochemical transformations in metabolic homeostasis. Due to the body’s inability to store these vitamins, they must be replenished daily. These vitamins function as coenzymes in numerous enzymatic processes and support a range of physiological cellular functions. An insufficient supply of B vitamins adversely affects mitochondrial metabolism of amino acids, glucose, and fatty acids through the citric acid cycle and the electron transport chain. This deficiency, in turn, impacts essential bodily systems, including the nervous and digestive systems (11). Vitamin B2, commonly known as riboflavin, is a crucial nutrient that primarily functions as a cofactor in the form of flavin adenine dinucleotide and flavin adenine mononucleotide in a wide array of enzymatic reactions across various life forms. Its significance in metabolic processes is underscored by its role in promoting electron transfer during biological oxidation-reduction reactions (12). A deficiency in riboflavin can adversely affect iron absorption, the metabolism of tryptophan, mitochondrial functionality, the health of the gastrointestinal tract, and the utilization of other vitamins, while it is also linked to various skin conditions (13).

Vitamin B2 (niacin) plays an important role in gastrointestinal health. Studies have shown that vitamin B2 can affect gastrointestinal function and health through a variety of mechanisms. Research indicates that vitamin B2 may play a role in regulating inflammatory processes within the gastrointestinal system, which in turn could affect bowel movement and the consistency of stool (14, 15). Furthermore, vitamin B2 has been correlated with particular bacterial taxa recognized as indicator species, such as *Fecalibacterium prausnitzii* and *Roseburia intestinalis*, which are essential for maintaining gut health, primarily through the synthesis of butyrate (16, 17). In poultry studies, the supplementation of vitamin B2 significantly regulated the cecal microbiota, increasing the abundance of health-promoting bacterial populations such as *Bifidobacteria*, and promoting the production of butyrate, a health-promoting metabolite, in the cecal environment (18). In addition, there was a significant negative correlation between vitamin B2 intake and *Helicobacter pylori* infection. Increased dietary intake of vitamin B2 may be associated with a lower seropositivity rate of *H. pylori*, suggesting that vitamin B2 may have a protective effect on gastric health (19). Vitamin B2 may also exert neuroprotective effects by inhibiting glutamate release from nerve endings, which may affect the neural regulation of the gastrointestinal tract. This inhibition is associated with a decrease in voltage-dependent calcium channel activity (20).

Considering the insufficient research on the potential benefits of vitamin B2 for intestinal health and its relationship with constipation, there is an evident need for comprehensive research in this domain. Therefore, this study evaluated the association between vitamin B2 and constipation in adults in the United States.

## 2 Materials and methods

### 2.1 Study population

The National Health and Nutrition Examination Survey (NHANES) is a comprehensive study conducted by the National Center for Health Statistics, which operates under the auspices of the Centers for Disease Control and Prevention (CDC) in the United States. This survey is designed to gather data from a representative cross-section of the American populace. The structure of the questionnaire employs stratification, a multistage design, and a probabilistic sampling methodology. Individuals taking part in the study undergo interviews that cover demographic, socio-economic, and health-related aspects at mobile medical examination facilities. This initial phase is followed by an extensive medical examination and laboratory evaluations. All participants provided their written informed consent, and the study protocol received clearance from the Ethics Review Board of the CDC.

**FIGURE 1:**
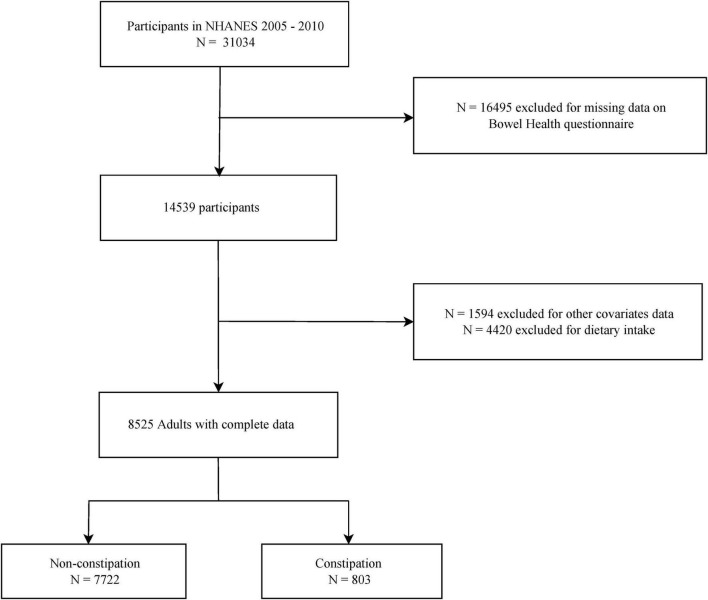
Flow diagram of study cohort selection from NHANES: 2005–2010.

NHANES cycles from 2005 to 2010 included a sample of 31,034 individuals. To maintain the integrity of the data and to specifically target the relevant adult demographic for our investigation, a set of exclusion criteria was systematically applied. This study excluded participants who were younger than 20 years of age or had missing information on bowel health questionnaire (*n* = 16,495), dietary intake (*n* = 4,420), or other covariates (*n* = 1,594). Consequently, a total of 8,525 individuals were identified as eligible participants for the present study. The enrollment process of these participants is depicted in Figure 1, which presents a flowchart detailing the selection process.

### 2.2 Variables

#### 2.2.1 Assessment of constipation

The definition of constipation is determined by referring to the self-reported defecation frequency or by assessing the consistency of the stool through the Bristol Stool Pattern Scale (BSFS) (21, 22). Previous studies have indicated a weak association between stool consistency and the frequency of defecation; however, the current investigation seeks to amalgamate these two variables to provide a more thorough assessment of constipation. Data regarding bowel habits were gathered from three cycles of the National Health and Nutrition Examination Survey (NHANES) conducted between 2005 and 2010, utilizing the Bristol Stool Form Scale (BSFS) for evaluation. This scale features cards displaying a range of colored images and descriptions that delineate seven distinct types of stools, which were employed to gauge the consistency of stools. Based on the Bristol Stool Form Scale, stool types 1 (identified as hard, nut-like lumps) and 2 (sausage-shaped yet lumpy) are classified as indicative of constipation, while stool types 3 through 7 are linked to non-constipated conditions. The assessment of defecation frequency was conducted by asking participants, “How often do you typically have bowel movements each week?” In this study, the diagnostic criteria for constipation comprised: (1) BSFS type I-II stool consistency, or (2) bowel movement frequency < 3 episodes weekly. Participants fulfilling ≥ 1 criterion were assigned to the constipation cohort (*n* = 803), with others constituting the non-constipated control group (*n* = 7,722).

#### 2.2.2 Assessment of vitamin B2 intake

Dietary vitamin B2 quantification employed duplicate 24 h dietary recalls administered non-consecutively. Initial assessments were conducted via face-to-face interviews, with telephone-based follow-up completed 3–10 days post-baseline. Consequently, in instances where an individual provided both 24 h dietary recalls, we calculated the average intake of vitamin B2 based on the data from these two recalls. In cases where only one recall was completed, we relied solely on the information gathered from the initial 24 h dietary recall. Dietary energy consumption was estimated using the United States Food and Nutrient Database for Dietary Studies. Absolute vitamin B2 intake was energy-adjusted via residual regression modeling to account for within-person variability (23). Participants in this dietary survey were asked to assess their food intake from the previous day by indicating whether it was “much less than usual,” “usual” or “much more than usual.” The study included only participants who answered “usual” (24). Considering the effects of dietary fiber and water intake on constipation and other B vitamins, they were included as covariates.

#### 2.2.3 Covariates

We identified variables that could serve as potential covariates, informed by prior research findings. The variables included in our analysis were age, gender, educational level, race/ethnicity, poverty-to-income ratio (PIR), body mass index (BMI), total fiber intake (g), water intake (including total tap water and total bottled water intake, g), as well as smoking and alcohol consumption habits, along with the status of diabetes and hypertension. NHANES classified race/ethnicity into four distinct categories: non-Hispanic white, non-Hispanic black, Mexican American, and other. Education level was classified into three categories: less than high school, high school or equivalent, and college or higher. PIR was stratified into three classifications: less than 1.3, ranging from 1.3 to 3.5, and 3.5 or greater, and ≥ 3.5 was categorized as follows: under 25, between 25 and 30, and exceeding 30 kg/m^2^. Regarding smoking habits, individuals were categorized as either non-smokers or smokers. A moderate alcohol consumer is characterized as a male who ingests fewer than two alcoholic beverages daily or a female who consumes less than one daily. A heavy alcohol consumer is identified as a male who drinks two or more beverages per day or a female who has one or more drinks each day. Diabetes was identified through the use of diabetes medication or insulin, self-reported history, fasting glucose levels over 7 mmol/L, glycohemoglobin A1c levels above 6.5%, random blood glucose levels of 11.1 mmol/L or higher, or 2 h OGTT blood glucose levels of 11.1 mmol/L or higher. Hypertension was identified based on three criteria: current antihypertensive treatment, a healthcare professional’s diagnosis, or an average blood pressure of ≥ 140/90 mmHg.

### 2.3 Statistical analysis

In accordance with the analytic guidelines established by the National Health and Nutrition Examination Survey (NHANES), the analyses incorporated sample weights, stratification, and clustering techniques to appropriately address the complexities associated with the survey design. The baseline characteristics categorized by quartiles of vitamin B2 intake were reported as weighted means accompanied by standard deviations (SD) for continuous variables, while categorical variables were presented as weighted percentages with their respective SD. In this investigation, Vitamin B2 intake was segmented into quartiles, designating the lowest quartile as the reference category. The chi-square test was employed for categorical variables, and Analysis of Variance (ANOVA) was utilized for continuous variables. Trend tests were conducted to evaluate the trend of the quartiles (*P* for trend). A weighted logistic regression model was applied to evaluate the relationship between vitamin B2 intake and the incidence of constipation. Model 1 represented the unadjusted analysis, whereas Model 2 included adjustments for demographic variables including age, sex, educational attainment, race/ethnicity, and Poverty Income Ratio (PIR). Model 3 further adjusted for Body Mass Index (BMI), smoking status, alcohol consumption, total fiber intake, water intake, diabetes status, and hypertension status. Additionally, we performed restricted cubic spline (RCS) analyses to investigate potential non-linear associations between vitamin B2 intake and constipation. In many practical applications, the relationship between variables is not a simple linear one but rather a complex non-linear one. By using RCS, these complex relationships can be described more accurately, thereby improving the model’s fit. RCS has significant advantages in handling non-linear and periodic data, and can be combined with other statistical methods to enhance the interpretability and predictive accuracy of models.

The study also engaged in stratified and interaction analyses to assess possible variations in these associations across diverse demographic factors such as age groups, race/ethnicity, educational background, PIR, smoking habits, alcohol consumption, and obesity status (where obesity is defined as a BMI < 30 kg/m^2^ and non-obesity as a BMI ≥ 30 kg/m^2^). Statistical analyses were conducted using R version 4.4.2, with all statistical tests being two-tailed and a significance level established at *P* < 0.05.

## 3 Results

### 3.1 Characteristics of the participants

This study encompassed a total of 8,525 individuals from NHANES conducted between 2005 and 2010, with an average age of 51.15 years. The fundamental characteristics of the participants are outlined in Table 1. Those situated in the highest quartile of vitamin B2 intake exhibited a tendency to be younger, male, non-Hispanic white, smokers, and possessed lower educational level and BMI. The mean vitamin B2 consumption among the participants was recorded at 2.15 mg.

**TABLE 1:** Baseline characteristics stratified by quartile of vitamin B2 intake.

Variable	Quartile 1	Quartile 2	Quartile 3	Quartile 4	*P*-value
Age, years	47.18 (45.96, 48.39)	49.60 (48.46, 50.73)	48.70 (47.75, 49.64)	47.15 (45.91, 48.39)	0.001
BMI, kg/m^2^	28.66 (28.24, 29.08)	28.96 (28.47, 29.44)	28.74 (28.36, 29.13)	28.40 (28.00, 28.80)	0.13
Sex, %					< 0.001
Female	70.46 (68.29, 72.62)	60.75 (57.77, 63.74)	48.43 (45.87, 50.99)	31.88 (29.13, 34.63)	–
Male	29.54 (27.38, 31.71)	39.25 (36.26, 42.23)	51.57 (49.01, 54.13)	68.12 (65.37, 70.87)	–
Race/ethnicity, %					< 0.001
Non-Hispanic white	60.34 (54.82, 65.85)	76.15 (72.68, 79.61)	80.56 (76.82, 84.30)	85.01 (82.35, 87.67)	–
Non-Hispanic black	17.90 (15.05, 20.75)	9.05 (7.16, 10.93)	6.04 (4.67, 7.40)	4.80 (3.62, 5.98)	–
Mexican American	9.74 (7.11, 12.36)	6.51 (4.85, 8.17)	6.14 (4.32, 7.97)	4.48 (3.10, 5.86)	
Other race	12.03 (8.71, 15.34)	8.30 (6.31, 10.29)	7.26 (5.25, 9.28)	5.70 (4.37, 7.04)	
Educational level, %					< 0.001
Less than high school	22.88 (20.21, 25.55)	15.27 (12.97, 17.58)	13.10 (10.98, 15.22)	13.03 (11.16, 14.90)	–
High school or equivalent	26.80 (24.00, 29.61)	25.69 (23.16, 28.23)	23.27 (20.69, 25.85)	23.39 (20.53, 26.26)	–
College or more	50.32 (47.27, 53.37)	59.03 (55.57, 62.49)	63.63 (59.67, 67.58)	63.58 (59.98, 67.17)	–
Poverty to income ratio, %					< 0.001
< 1.3	27.28 (24.57, 29.99)	16.89 (14.56, 19.22)	13.16 (10.98, 15.34)	15.21 (13.26, 17.15)	–
1.3–3.49	39.73 (37.19, 42.27)	37.75 (34.30, 41.19)	33.86 (30.66, 37.07)	33.10 (29.85, 36.34)	–
≥ 3.5	32.99 (30.00, 35.97)	45.37 (41.55, 49.18)	52.98 (48.87, 57.08)	51.70 (48.48, 54.91)	–
Smoke, %					0.02
No	57.04 (53.65, 60.44)	54.16 (51.45, 56.87)	53.72 (50.19, 57.25)	50.20 (46.81, 53.58)	–
Yes	42.96 (39.56, 46.35)	45.84 (43.13, 48.55)	46.28 (42.75, 49.81)	49.80 (46.42, 53.19)	–
Alcohol intake, %					< 0.001
None	36.30 (32.91, 39.68)	28.13 (25.48, 30.78)	23.16 (20.37, 25.95)	23.39 (20.42, 26.36)	–
Low to moderate	45.25 (42.07, 48.43)	50.15 (47.43, 52.87)	56.59 (52.95, 60.23)	57.58 (53.74, 61.42)	–
Heavy	18.45 (15.90, 21.00)	21.72 (19.58, 23.86)	20.26 (17.61, 22.90)	19.03 (16.99, 21.07)	–
Diabetes, %					0.001
No	85.51 (83.10, 87.91)	85.39 (83.42, 87.37)	87.28 (85.43, 89.14)	90.00 (88.57, 91.42)	–
Yes	14.49 (12.09, 16.90)	14.61 (12.63, 16.58)	12.72 (10.86, 14.57)	10.00 (8.58, 11.43)	–
Constipation					< 0.001
No	86.01 (83.65, 88.37)	90.43 (88.99, 91.87)	92.38 (91.15, 93.61)	94.22 (92.73, 95.70)	–
Yes	13.99 (11.63, 16.35)	9.57 (8.13, 11.01)	7.62 (6.39, 8.85)	5.78 (4.30, 7.27)	–
Hypertension, %					0.13
No	60.04 (56.83, 63.25)	60.98 (57.96, 64.00)	63.51 (60.27, 66.76)	64.96 (61.30, 68.62)	–
Yes	39.96 (36.75, 43.17)	39.02 (36.00, 42.04)	36.49 (33.24, 39.73)	35.04 (31.38, 38.70)	–

Data are presented as mean (SD) or *n* (%). BMI, body mass index.

### 3.2 Association between vitamin B2 intake and constipation

Table 2 illustrates the findings derived from the logistic regression weighted model that explored the association between vitamin B2 consumption and the incidence of constipation. In the unadjusted model 1, those in the highest quartile of vitamin B2 intake exhibited a significantly reduced likelihood of experiencing constipation (OR = 0.38; 95% CI, 0.29–0.50) when compared to participants in the lowest quartile. This association persisted in model 2 (OR = 0.62; 95% CI, 0.46–0.83). In the fully adjusted model 3, individuals within the highest quartile of vitamin B2 intake continued to demonstrate a lower risk of constipation (OR = 0.63; 95% CI, 0.47–0.84) relative to those in the lowest quartile. Furthermore, a notable inverse relationship was observed between continuous vitamin B2 intake and constipation in model 3 (OR = 0.89, 95% CI, 0.81–0.98).

**TABLE 2:** Association between vitamin B2 and constipation.

	Case/*n*	OR (95% CI)		
Continuous		Model 1	Model2	Model 3
	803/8525	0.77 (0.69, 0.85)***	0.89 (0.81, 0.98)*	0.89 (0.81, 0.98)*
**Categories**				
Q1	287/2134	1.00 (reference)	1.00 (reference)	1.00 (reference)
Q2	201/2131	0.65 (0.49, 0.86)**	0.79 (0.58, 1.07)	0.81 (0.60, 1.10)
Q3	182/2129	0.51 (0.38, 0.67)***	0.71 (0.52, 0.98)*	0.73 (0.53, 1.01)
Q4	133/2131	0.38 (0.29, 0.50)***	0.62 (0.46, 0.83)**	0.63 (0.47, 0.84)**
*P* for trend	–	< 0.001	0.003	0.003

Model 1 was a crude model. Model 2 was adjusted for age, sex, race/ethnicity, education level and PIR. Model 3 was further adjusted for BMI, smoker, drinker, total fiber intake, water intake, diabetes status and hypertension status. BMI, body mass index; PIR, poverty to income ratio.

^*^P < 0.05;

^**^P < 0.01;

^***^P < 0.001.

**FIGURE 2:**
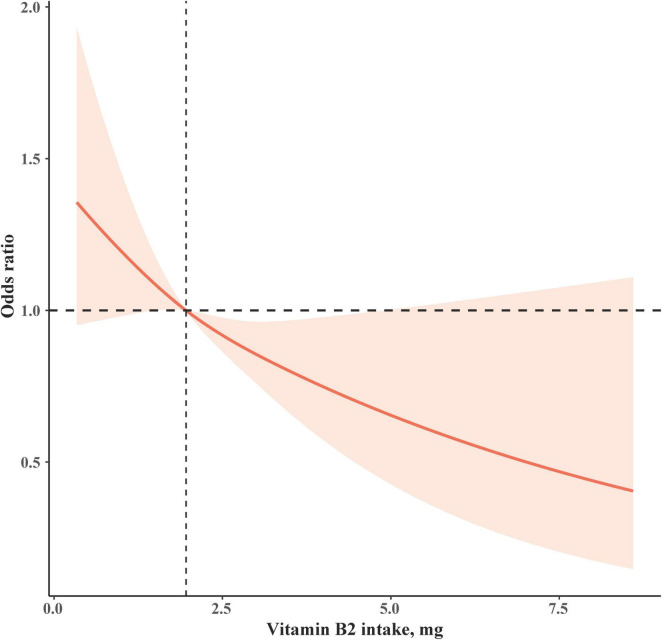
The association between vitamin B2 intake and constipation in the fully adjusted model. Adjustments included age, sex, race/ethnicity, education level, and PIR BMI, smoker, drinker, total fiber intake, water intake, diabetes status, and hypertension status. BMI, body mass index; PIR, poverty to income ratio.

In addition, we performed restricted cubic spline (RCS) analyses to further investigate the associations between vitamin B2 intake and constipation. In model 3, following full adjustments, a linear relationship was identified between vitamin B2 intake and constipation (*P*-non-linear = 0.7297, *P*-overall = 0.0224; Figure 2).

### 3.3 Subgroup analysis

The results of subgroup analyses to assess the association between vitamin B2 and constipation were presented in Table 3. This inverse association persisted in the subgroup analysis based on age group, sex, PIR, and in non-Hispanic white, as well as in participants currently smoking, with non-obesity. However, a significant interaction between vitamin B2 intake and alcohol consumption for constipation was observed (*P* interaction = 0.012).

**TABLE 3:** Subgroup analysis of the association between dietary vitamin B2 intake and constipation.

Subgroup	Quartile 1	Quartile 2	Quartile 3	Quartile 4	*P* interaction
**Age**					0.896
20–59 years	Ref	0.72 (0.52, 1.00)	0.70 (0.51, 0.98)	0.62 (0.43, 0.90)	
≥ 60 years	Ref	0.96 (0.61, 1.52)	0.75 (0.46, 1.24)	0.55 (0.35, 0.87)	
**Sex**					
Female	Ref	1.11 (0.86, 1.44)	0.78 (0.49, 1.19)	0.65 (0.31, 0.89)	0.184
Male	Ref	0.69 (0.41, 1.15)	0.51 (0.31, 0.85)	0.34 (0.19, 0.59)	
**Race/ethnicity**					
Non-Hispanic white	Ref	0.93 (0.67, 1.28)	0.67 (0.50, 0.91)	0.59 (0.38, 0.93)	0.067
Others	Ref	0.81 (0.54, 1.21)	0.74 (0.49, 1.12)	0.67 (0.43, 1.04)	
**Education level**					0.395
Less than high school	Ref	0.81 (0.49, 1.34)	0.75 (0.42, 1.35)	0.47 (0.25, 0.87)	
High school or above	Ref	0.84 (0.60, 1.16)	0.72 (0.53, 0.97)	0.64 (0.45, 0.91)	
**PIR**					0.287
< 2.5	Ref	0.84 (0.60, 1.19)	0.90 (0.62, 1.31)	0.63 (0.42, 0.95)	
≥ 2.5	Ref	0.71 (0.46, 1.08)	0.55 (0.35, 0.88)	0.51 (0.30, 0.87)	
**Smoking status**					0.197
No	Ref	0.93 (0.65, 1.35)	0.97 (0.65, 1.45)	0.68 (0.45, 1.05)	
Yes	Ref	0.78 (0.54, 1.12)	0.62 (0.40, 0.97)	0.57 (0.41, 0.77)	
**Alcohol consumption**					0.012
No	Ref	1.05 (0.66, 1.67)	1.03 (0.62, 1.69)	1.04 (0.62, 1.74)	
Yes	Ref	0.74 (0.52, 1.04)	0.59 (0.44, 0.78)	0.48 (0.33, 0.70)	
**Obesity status**					0.736
Obesity	Ref	1.04 (0.63, 1.70)	0.87 (0.50, 1.52)	0.59 (0.33, 1.08)	
Non-obesity	Ref	0.75 (0.55, 1.04)	0.72 (0.49, 1.06)	0.64 (0.46, 0.91)	

Adjustments included age, sex, race/ethnicity, education level, and PIR BMI, smoker, drinker, total fiber intake, water intake, diabetes status, and hypertension status. BMI, body mass index; PIR, poverty to income ratio.

## 4 Discussion

In this comprehensive nationwide cross-sectional study utilizing data from the NHANES 2005–2010, we explored the relationship between dietary intake of vitamin B2 and the prevalence of constipation. The weighted logistic regression analysis revealed a significant inverse correlation (OR = 0.38; 95% CI, 0.29–0.50), indicating that higher vitamin B2 consumption is associated with lower risk of constipation. Furthermore, the restricted cubic spline analysis demonstrated a linear correlation between vitamin B2 intake and constipation incidence. Notably, subgroup analyses uncovered an interaction effect between alcohol consumption and the association of vitamin B2 intake with constipation, revealing that individuals who currently consume alcohol showed a significantly stronger protective influence from dietary vitamin B2.

The initial approach to managing chronic constipation generally involves modifications to diet and lifestyle. Consequently, there is an urgent requirement to intensify research initiatives in this domain to improve the handling of this condition. Throughout the years, researchers have increasingly documented possible correlations between chronic constipation and dietary influences by conducting thorough examinations of the NHANES database (25–29). The study by Jiang et al. demonstrated that the intake of dietary flavonoids is linked to a reduced risk of constipation among adults in the United States, with a notably consistent association observed for anthocyanins (30). Similarly, research by Zhao et al. identified a positive correlation between the dietary inflammatory index and constipation, highlighting its potential importance in assessing chronic constipation conditions (31). Cai et al. (6) have elucidated a correlation between dietary vitamin E intake and the occurrence of chronic constipation among adults in the United States. Collectively, these findings underscore the significance of integrating a diverse range of dietary indicators in the evaluation of chronic constipation.

Riboflavin, a constituent of the vitamin B complex, is found in a variety of plant and animal-based foods, including enriched green vegetables, nuts, meat, milk, eggs, and flour (32). Since humans lack the ability to produce riboflavin internally, it is necessary for them to acquire it through their diet or through supplementation (33). This vitamin plays a crucial role as a fundamental element of coenzymes that participate in processes such as cell growth, energy metabolism, and the breakdown of fat tissue (17).

The possible correlation between vitamin B2 consumption and constipation encompasses several aspects that warrant further academic investigation. The generation of reactive oxygen species (ROS) and the ensuing oxidative stress are pivotal in the pathogenesis and progression of numerous diseases, including those affecting the intestines. An overabundance of ROS can inflict damage on cellular structures and molecules, such as lipids, proteins, and DNA, which ultimately contributes to the development of intestinal diseases (34–36). Oxidative stress has the potential to compromise the functionality of interstitial cells of cajal and induce apoptosis, thereby impairing intestinal motility and potentially contributing to the pathogenesis of constipation (37). Riboflavin, often neglected as an antioxidant, may exert its antioxidant effects either through its conversion from the reduced to the oxidized form or by serving as a crucial element within the glutathione redox cycle (38). Studies have shown that 3 weeks of riboflavin supplementation resulted in a reduction in systemic oxidative stress, mixed anti-inflammatory effects, and improved clinical symptoms by changing the composition of the gut flora in Crohn’s patients (39). In a study on broiler chickens, vitamin B2 supplementation significantly increased the abundance of health-promoting bacteria in the cecum, such as *Bifidobacteria*, and promoted the production of health-promoting metabolites like butyrate (18). Further research is needed to confirm the relationship between riboflavin and oxidative stress, gut microbiota and constipation.

Prior research has shown that riboflavin possesses anti-inflammatory and antioxidant properties in animal models afflicted with Crohn’s disease (25, 26, 40). Additional experimental investigations involving animal models have further indicated that riboflavin showcases anti-inflammatory characteristics, as evidenced by a decrease in the synthesis of pro-inflammatory cytokines such as tumor necrosis factor-alpha and interleukin-6 (17). These anti-inflammatory attributes may play a significant role in safeguarding the intestinal mucosa, consequently enhancing overall intestinal health and functionality.

Furthermore, vitamin B2 may influence gut health by regulating the function and composition of gut microbiota. Research indicated vitamin B2 can affect the metabolic activities and bacterial composition of gut microorganisms, thereby positively impacting host health (41). For example, vitamin B2 may maintain the equilibrium of the gut microbiome by fostering the proliferation of beneficial bacterial populations while simultaneously suppressing the growth of detrimental bacteria. A systematic review and meta-analysis investigating the effects of vitamin or mineral supplementation on chronic constipation in adults demonstrated that magnesium oxide supplementation significantly enhanced various constipation-related outcomes. This improvement is attributed to its mechanism of action, which involves water retention in the intestinal lumen, resulting in fecal expansion and softening (42). Compared to the control group, the likelihood of a positive treatment response increased by 332%. It has been hypothesized that vitamin C supplementation may ameliorate symptoms of constipation. This hypothesis is derived from limited evidence suggesting that high doses of vitamin C can induce diarrhea, potentially exerting a laxative effect that could be advantageous in the management of constipation (43). A case-control study has shown that vitamin D deficiency is associated with chronic functional constipation caused by intestinal motility disorders (44). And vitamin B2 may affect constipation through biological mechanisms such as oxidative stress and regulation of the intestinal flora. The consumption of alcohol is known to adversely affect metabolic processes and nutrient absorption, particularly impairing the uptake of various B vitamins. Subgroup analysis revealed an interaction effect of alcohol consumption on the relationship between vitamin B2 intake and constipation. Current drinkers may be associated with a lower risk of constipation. This might be due to the following several biological mechanisms. Alcohol consumption has been shown to augment the secretion of gastric acid and digestive fluids, leading to an elevated water content in the intestinal lumen. This physiological response prevents excessive desiccation of the stool and facilitates regular bowel movements (45). The maintenance of intestinal fluid homeostasis is crucial for ensuring normal stool consistency. Consequently, moderate alcohol intake may contribute to the retention of sufficient intestinal moisture, thereby diminishing the risk of constipation (46). Moreover, alcohol consumption has been shown to modify the composition of the gut microbiota, notably by increasing the prevalence of pathogenic bacteria while decreasing the abundance of beneficial microbial populations. This dysbiosis can alter the intestinal milieu, potentially impairing the absorption of nutrients and fluids, thereby leading to an increase in bowel movements (47, 48). Subramanian and colleagues demonstrated in an animal experiment that long-term exposure to ethanol through the Lieber DeCarli ethanol diet would inhibit the absorption of vitamin B2 in the intestines of male Wistar rats (49). At the molecular level, alcohol reduced the protein expression of the vitamin B2 transporter and the levels of heteronuclear RNA (hnRNA), the latter serving as an indicator of transcriptional activity. Further research is necessary to enhance the understanding of the inhibitory effects of alcohol on vitamin B2 absorption and its relationship to constipation.

This study has several limitations. Firstly, NHANES collected cross-sectional data, precluding the determination of causal relationships between vitamin B2 intake and constipation. Secondly, self-reported dietary 24 h recall data per person may be limited to characterizing diet over a person’s lifespan and are subject to measurement errors due to large day-to-day variations in food intake. Thirdly, despite adjusting for a comprehensive array of potential confounders, the influence of unmeasured or residual confounding factors cannot be entirely excluded. Fourthly, excluding individuals with incomplete data may lead to potential selective bias.

## 5 Conclusion

In summary, our study reveals a negative correlation between dietary vitamin B2 consumption and chronic constipation among the general adult populace. Future research necessitates large-scale, multicenter, and high-quality studies to validate these findings and to further elucidate the molecular mechanisms underlying the effects of vitamin B2 on constipation. Healthcare professionals are advised to prioritize the promotion of a well-balanced diet as an initial therapeutic approach, preceding medical interventions.

## Data Availability

Publicly available datasets were analyzed in this study. This data can be found here: The National Health and Nutrition Examination Survey dataset is publicly available at the National Center for Health Statistics of the Center for Disease Control and Prevention (https://wwwn.cdc.gov/nchs/nhanes/default.aspx).

## References

[1] Higgins PD Johanson JF. Epidemiology of constipation in North America: A systematic review. *Am J Gastroenterol.* (2004) 99:750–9. 10.1111/j.1572-0241.2004.04114.x 15089911

[2] Belsey J Greenfield S Candy D Geraint M. Systematic review: Impact of constipation on quality of life in adults and children. *Aliment Pharmacol Ther.* (2010) 31:938–49. 10.1111/j.1365-2036.2010.04273.x 20180788

[3] Suares NC Ford AC. Prevalence of, and risk factors for, chronic idiopathic constipation in the community: Systematic review and meta-analysis. *Am J Gastroenterol.* (2011) 106:1582–1591; quiz 1581, 1592. 10.1038/ajg.2011.164 21606976

[4] Talley NJ Lasch KL Baum CL. A gap in our understanding: Chronic constipation and its comorbid conditions. *Clin Gastroenterol Hepatol.* (2009) 7:9–19. 10.1016/j.cgh.2008.07.005 18829389

[5] Oh SJ Fuller G Patel D Khalil C Spalding W Nag A Chronic constipation in the United States: Results from a population-based survey assessing healthcare seeking and use of pharmacotherapy. *Am J Gastroenterol.* (2020) 115:895–905. 10.14309/ajg.0000000000000614 32324606 PMC7269025

[6] Cai J Li D Xie R Yu X Wu Y Sun F Association between dietary vitamin E intake and constipation: NHANES 2005-2010. *Front Nutr.* (2024) 11:1426280. 10.3389/fnut.2024.1426280 39229590 PMC11368839

[7] Du W Lu L Liu Y Yan Y La R Wu Q The association between dietary vitamin B1 intake and constipation: A population-based study. *BMC Gastroenterol.* (2024) 24:171. 10.1186/s12876-024-03255-2 38760704 PMC11100033

[8] Zhao X Wang L Quan L. Association between dietary phosphorus intake and chronic constipation in adults: Evidence from the national health and nutrition examination survey. *BMC Gastroenterol.* (2023) 23:24. 10.1186/s12876-022-02629-8 36694113 PMC9875444

[9] Dupont C Hébert G. Magnesium sulfate-rich natural mineral waters in the treatment of functional constipation-a review. *Nutrients.* (2020) 12:2052. 10.3390/nu12072052 32664341 PMC7400933

[10] Wang C Zhang L Li L. Association between selenium intake with chronic constipation and chronic diarrhea in adults: Findings from the national health and nutrition examination survey. *Biol Trace Elem Res.* (2021) 199:3205–12. 10.1007/s12011-020-02451-x 33095434

[11] Kennedy DO. B vitamins and the brain: Mechanisms, dose and efficacy–a review. *Nutrients.* (2016) 8:68. 10.3390/nu8020068 26828517 PMC4772032

[12] Alam MM Iqbal S Naseem I. Ameliorative effect of riboflavin on hyperglycemia, oxidative stress and DNA damage in type-2 diabetic mice: Mechanistic and therapeutic strategies. *Arch Biochem Biophys.* (2015) 584:10–9. 10.1016/j.abb.2015.08.013 26319175

[13] Thakur K Tomar SK Singh AK Mandal S Arora S. Riboflavin and health: A review of recent human research. *Crit Rev Food Sci Nutr.* (2017) 57:3650–60. 10.1080/10408398.2016.1145104 27029320

[14] Zhang WW Thakur K Zhang JG Wei ZJ. Riboflavin ameliorates intestinal inflammation via immune modulation and alterations of gut microbiota homeostasis in DSS-colitis C57BL/6 mice. *Food Funct.* (2024) 15:4109–21. 10.1039/d4fo00835a 38597225

[15] Zhu YY Thakur K Feng JY Zhang JG Hu F Cespedes-Acuña CL Riboflavin bioenriched soymilk alleviates oxidative stress mediated liver injury, intestinal inflammation, and gut microbiota modification in B(2) depletion-repletion mice. *J Agric Food Chem.* (2022) 70:3818–31. 10.1021/acs.jafc.2c00117 35302755

[16] Olfat N Ashoori M Saedisomeolia A. Riboflavin is an antioxidant: A review update. *Br J Nutr.* (2022) 128:1887–95. 10.1017/S0007114521005031 35115064

[17] Suwannasom N Kao I Pruß A Georgieva R Bäumler H. Riboflavin: The health benefits of a forgotten natural vitamin. *Int J Mol Sci.* (2020) 21:950. 10.3390/ijms21030950 32023913 PMC7037471

[18] Biagi E Mengucci C Barone M Picone G Lucchi A Celi P Effects of vitamin B2 supplementation in broilers microbiota and metabolome. *Microorganisms.* (2020) 8:1134. 10.3390/microorganisms8081134 32727134 PMC7464963

[19] Huang Y Ao T Zhen P Hu M. Correlation between dietary vitamin B2 intake and *Helicobacter pylori* infection in US adults: A cross-sectional study. *J Health Popul Nutr.* (2025) 44:76. 10.1186/s41043-025-00815-4 40075545 PMC11905576

[20] Wang S Wu W Yang F Hsu G Huang C. Vitamin B2 inhibits glutamate release from rat cerebrocortical nerve terminals. *Neuroreport.* (2008) 19:1335–8. 10.1097/WNR.0b013e32830b8afa 18695519

[21] Markland AD Palsson O Goode PS Burgio KL Busby-Whitehead J Whitehead WE. Association of low dietary intake of fiber and liquids with constipation: Evidence from the national health and nutrition examination survey. *Am J Gastroenterol.* (2013) 108:796–803. 10.1038/ajg.2013.73 23567352 PMC3786707

[22] Wang DC Peng XF Chen WX Yu M. The association of moisture intake and constipation among us adults: Evidence from NHANES 2005-2010. *BMC Public Health.* (2025) 25:399. 10.1186/s12889-025-21346-x 39891106 PMC11783823

[23] Willett WC Howe GR Kushi LH. Adjustment for total energy intake in epidemiologic studies. *Am J Clin Nutr.* (1997) 65:1220S–8S. 10.1093/ajcn/65.4.1220S 9094926

[24] Zheng L Sun J Yu X Zhang D. Ultra-processed food is positively associated with depressive symptoms among United States adults. *Front Nutr.* (2020) 7:600449. 10.3389/fnut.2020.600449 33385006 PMC7770142

[25] Menezes RR Godin AM Rodrigues FF Coura G Melo I Brito A Thiamine and riboflavin inhibit production of cytokines and increase the anti-inflammatory activity of a corticosteroid in a chronic model of inflammation induced by complete Freund’s adjuvant. *Pharmacol Rep.* (2017) 69:1036–43. 10.1016/j.pharep.2017.04.011 28958614

[26] Steinert RE Sadaghian Sadabad M Harmsen HJ Weber P. The prebiotic concept and human health: A changing landscape with riboflavin as a novel prebiotic candidate. *Eur J Clin Nutr.* (2016) 70:1348–53. 10.1038/ejcn.2016.119 27380884

[27] Wei S Yu S Lan Y Jia Y. Association between the composite dietary antioxidant index and constipation: Evidence from NHANES 2005-2010. *PLoS One.* (2024) 19:e0311168. 10.1371/journal.pone.0311168 39331658 PMC11432863

[28] Kong W Sheng W Zheng Y. Modification of the association between coffee consumption and constipation by alcohol drinking: A cross-sectional analysis of NHANES 2007-2010. *PLoS One.* (2024) 19:e0311916. 10.1371/journal.pone.0311916 39453914 PMC11508157

[29] Wang B Liu C Guo Z Li R Wang Y Dong C Association of dietary inflammatory index with constipation: Evidence from the national health and nutrition examination survey. *Food Sci Nutr.* (2024) 12:2122–30. 10.1002/fsn3.3914 38455207 PMC10916608

[30] Jiang C Luo J Shao Y. Evaluating the relationship between dietary flavonoids intake and constipation incidence in the general US population. *BMC Gastroenterol.* (2024) 24:455. 10.1186/s12876-024-03551-x 39696041 PMC11654161

[31] Zhao X Wang X Quan L. Association between dietary inflammatory index and energy-adjusted dietary inflammatory index and constipation in US adults. *BMC Gastroenterol.* (2024) 24:235. 10.1186/s12876-024-03307-7 39060983 PMC11282795

[32] Powers HJ. Riboflavin (vitamin B-2) and health. *Am J Clin Nutr.* (2003) 77:1352–60. 10.1093/ajcn/77.6.1352 12791609

[33] Liu M Zhou C Zhang Z Li Q He P Zhang Y Inverse association between riboflavin intake and new-onset hypertension: A nationwide cohort study in China. *Hypertension.* (2020) 76:1709–16. 10.1161/HYPERTENSIONAHA.120.16211 33131313

[34] Rosa V Lucia P Martina C Carola S Paola M. The impact of oxidative stress in human pathology: Focus on gastrointestinal disorders. *Antioxidants.* (2021) 10:201. 10.3390/antiox10020201 33573222 PMC7910878

[35] Peng X Yi X Deng N Liu J Tan Z Cai Y. Zhishi Daozhi decoction alleviates constipation induced by a high-fat and high-protein diet via regulating intestinal mucosal microbiota and oxidative stress. *Front Microbiol.* (2023) 14:1214577. 10.3389/fmicb.2023.1214577 37789856 PMC10544343

[36] Felemovicius I Bonsack ME Baptista ML Delaney JP. Intestinal radioprotection by vitamin E (alpha-tocopherol). *Ann Surg.* (1995) 222:504–508; discussion 508–510. 10.1097/00000658-199522240-00008 7574930 PMC1234882

[37] Yao Z Fu S Ren B Ma L Sun D. Based on network pharmacology and gut microbiota analysis to investigate the mechanism of the laxative effect of pterostilbene on loperamide-induced slow transit constipation in mice. *Front Pharmacol.* (2022) 13:913420. 10.3389/fphar.2022.913420 35652049 PMC9148975

[38] Ashoori M Saedisomeolia A. Riboflavin (vitamin B2) and oxidative stress: A review. *Br J Nutr.* (2014) 111:1985–91. 10.1017/S0007114514000178 24650639

[39] von Martels J Bourgonje AR Klaassen M Alkhalifah H Sadaghian Sadabad M Vich Vila A Riboflavin supplementation in patients with Crohn’s disease [The RISE-UP study]. *J Crohns Colitis.* (2020) 14:595–607. 10.1093/ecco-jcc/jjz208 31873717 PMC7303596

[40] Levit R Savoy de Giori G de Moreno de LeBlanc A LeBlanc JG. Effect of riboflavin-producing bacteria against chemically induced colitis in mice. *J Appl Microbiol.* (2018) 124:232–40. 10.1111/jam.13622 29080295

[41] Pham VT Fehlbaum S Seifert N Richard N Bruins MJ Sybesma W Effects of colon-targeted vitamins on the composition and metabolic activity of the human gut microbiome- a pilot study. *Gut Microbes.* (2021) 13:1–20. 10.1080/19490976.2021.1875774 33615992 PMC7899684

[42] van der Schoot A Creedon A Whelan K Dimidi E. The effect of food, vitamin, or mineral supplements on chronic constipation in adults: A systematic review and meta-analysis of randomized controlled trials. *Neurogastroenterol Motil.* (2023) 35:e14613. 10.1111/nmo.14613 37243443

[43] Miller DR. Vitamin excess and toxicity. *Nutr Toxicol.* (1982) 1:81–133. 10.1016/B978-0-12-332601-0.50008-5

[44] Panarese A Pesce F Porcelli P Riezzo G Iacovazzi P Leone C Chronic functional constipation is strongly linked to vitamin D deficiency. *World J Gastroenterol.* (2019) 25:1729–40. 10.3748/wjg.v25.i14.1729 31011257 PMC6465937

[45] Chen W Peng X Yu M Wang D. Daily alcohol intake and its negative association with constipation based on NHANES data 2005-2010. *Sci Rep.* (2025) 15:10021. 10.1038/s41598-025-91899-9 40122926 PMC11930976

[46] Asher G Sassone-Corsi P. Time for food: The intimate interplay between nutrition, metabolism, and the circadian clock. *Cell.* (2015) 161:84–92. 10.1016/j.cell.2015.03.015 25815987

[47] Phillip AE Stefan JG Robin MV Christopher BF Ali K. The gastrointestinal microbiome: Alcohol effects on the composition of intestinal microbiota. *Alcohol Res Curr Rev.* (2015) 37:223–36. 10.35946/arcr.v37.2.07 PMC4590619 26695747

[48] Capurso G Lahner E. The interaction between smoking, alcohol and the gut microbiome. *Best Pract Res Clin Gastroenterol.* (2017) 31:579–88. 10.1016/j.bpg.2017.10.006 29195678

[49] Subramanian V Subramanya S Ghosal A Said H. Chronic alcohol feeding inhibits physiological and molecular parameters of intestinal and renal riboflavin transport. *Am J Physiol Cell Physiol.* (2013) 305:C539–46. 10.1152/ajpcell.00089.2013 23804199 PMC3761153

